# A Framework for the Development of Context-Adaptable User Interfaces for Ubiquitous Computing Systems

**DOI:** 10.3390/s16071049

**Published:** 2016-07-07

**Authors:** Gervasio Varela, Alejandro Paz-Lopez, Jose A. Becerra, Richard Duro

**Affiliations:** 1 Mytech Ingeniería Aplicada S.L., Ferrol 15403, Spain; alejandro.paz@mytechia.com; 2 Integrated Group for Engineering Research, University of A Coruña, Ferrol 15403, Spain; ronin@udc.es (J.A.B.); richard@udc.es (R.D.)

**Keywords:** ubiquitous computing, user interface, human-computer interface, software engineering, ambient intelligence

## Abstract

This paper addresses the problem of developing user interfaces for Ubiquitous Computing (UC) and Ambient Intelligence (AmI) systems. These kind of systems are expected to provide a natural user experience, considering interaction modalities adapted to the user abilities and preferences and using whatever interaction devices are present in the environment. These interaction devices are not necessarily known at design time. The task is quite complicated due to the variety of devices and technologies, and the diversity of scenarios, and it usually burdens the developer with the need to create many different UIs in order to consider the foreseeable user-environment combinations. Here, we propose an UI abstraction framework for UC and AmI systems that effectively improves the portability of those systems between different environments and for different users. It allows developers to design and implement a single UI capable of being deployed with different devices and modalities regardless the physical location.

## 1. Introduction

In Ubiquitous Computing (UC) and Ambient Intelligence (AmI) systems the interaction with the user is a critical aspect [1]. These systems are strongly related to people and their needs, providing functionalities assisting them in their daily life wherever they may be located. They are expected to provide a natural user experience, in which the UI blends in with the environment [2,3], reducing interaction to the minimum through interaction devices and modalities adapted to the user needs and abilities. Thus, to construct this kind of user experience, UC and AmI developers need to build the user interfaces (UIs) of their systems relying on a combination of different devices distributed throughout the physical environment [4,5]. These devices usually correspond to different types of systems coming from different manufacturers and technologies. Additionally, adapted and natural user interfaces require different interaction modalities (voice, touch, tangible UIs, gestures, etc.) and these must be chosen depending on the type of user or the environmental conditions. For example, a voice-based UI may be a good option for a blind user in their home, but is probably a poor choice for a deaf user, or even for the blind user in a noisy environment like a crowded street.

Developers, and their code, need to handle all this heterogeneity. This is usually addressed through the design and implementation of different UIs for each combination of environment and user, thus greatly increasing the complexity and costs of developing UC and AmI systems.

Research into these problems has been carried out mainly within two important, but generally unrelated fields. On the one hand the field of Physical User Interfaces (PUIs) has been producing solutions for many years to facilitate the use of physical devices to interact with the user. Examples are the iStuff [6] or EIToolKit [7] frameworks. However, these frameworks lack high-level abstraction capabilities. This implies that it is necessary to modify the code to port an UI to a new environment where different devices and modalities are required. On the other hand, in the field of UI adaptation solutions, like Egoki [8], the Multi-Access Service Platform [9] or the Personal Universal Controller [10], most researchers have concentrated on generating tailored graphical user interfaces for the services or devices available in a smart environment. The focus of these frameworks is on graphical or voice-based UIs, but they do not usually provide support for the interaction with the user through a combination of multi-modal physical devices that are distributed in the environment.

This paper addresses the general problem through a UI abstraction technology for the development of UC and AmI systems that effectively improves the portability of those systems between different environments. This is achieved by increasing the decoupling between the developers, their code, and the final modalities and physical devices used to implement the UI for a particular combination of user and environment characteristics. The objective is to allow the developers to design and implement a single UI, and then be able to easily deploy it using different devices and modalities in different physical locations by introducing a new development framework for Ubiquitous Computing UI development, called Dandelion, that greatly increases the decoupling between the business logic, the UI control logic, and the interaction resources.

The rest of the paper is organized as follows: Section 2 provides a review of related work in the field. In Section 3 we provide an introduction to the Dandelion framework including initial examples of the implementation of particular characteristics using it. Section 4 is devoted to a comparative analysis of the solution, both from a qualitative and a quantitative point of view. Finally, in Section 5 we present some conclusions.

## 2. Related Work

It is well recognized that one of the obstacles in the path towards achieving truly ubiquitous systems capable of operating in a diversity of scenarios, while maintaining an acceptable level of user experience, is that it is quite difficult to foresee, and provide support for, the wide range of circumstances, settings, technologies and devices (interaction resources) required to build UIs capable of providing adequate user interaction in a diversity of contexts of use.

Here we address this problem by increasing the level of decoupling between the UI definition, the UI logic and the particular realization of the UI for a specific scenario (modalities, devices, user characteristics, environment characteristics, etc.). The goal is to improve the portability of ubiquitous computing systems, so that they can be more easily adaptable to different contexts.

The portability and adaptability of user interfaces has been an important research topic for the UI community during the last years, specially due to the proliferation of multiple hardware platforms (PC, smartphones, tablets, smart TVs, etc.) and the necessity of providing UIs adapted to people with physical or cognitive disabilities. The result is the concept of plastic user interfaces [11].

The term plasticity of UIs refers to user interfaces that can be adapted, either automatically or manually, to changes in the context, while preserving the utility and usability of a system at acceptable levels. Plastic UIs help in reducing the development cost of applications because they allow the reutilization of the same application and UI code with different devices and in a variety of environments. Plastic UIs go beyond the code independence of the platform of virtual machines, and beyond the abstraction from graphic toolkits of Interface Definition Languages (IDLs). Plastic UIs are expected to provide independence from the context of use, allowing developers to modify and adapt the mode of interaction and even change the physical shape of the UI, preferably in an autonomous way, based on the interaction resources available and the state and characteristics of the user and environment where the interaction takes place.

Much work has been dedicated to advancing in the addition of plasticity capabilities to traditional user interfaces, like graphical and voice-based UIs. Nevertheless, we think that Ubiquitous Computing (UC) user interfaces present a series of characteristics that make them quite different from traditional interfaces.

First, compared to traditional UIs, UC UIs are exposed to a much broader range of technologies, APIs and interaction modalities because they are often built as distributed physical user interfaces (DPUIs) [4,5,6] using many different devices, each one of them specifically selected to match one particular context of use.

Second, UC systems are expected to be physically integrated in the environment and use the environment itself to interact with its users. Furthermore, they are expected to be ubiquitous, so they must provide their functionality in multiple physical locations. Because of that, the multiple devices that make up an UC UI tend to be physically scattered throughout one or more physical environments.

And third, due to the natural interaction constraints and the use of a combination of multiple interaction modalities, an UC UI can be dramatically affected by context changes. A new user, a change of location, or a change in the environment characteristics can even render an UI unusable.

For example, in a new location, the devices and modalities available can be completely different from the previous one. A new user may need different modalities (imagine the conflicting requirements of a deaf user as compared to a blind user). And even a change in environment characteristics, like lighting or movement, can affect the functionality of the UI.

As can be seen, plasticity can be hard to support in Ubiquitous Computing UIs, but, nevertheless, it is a key requirement in order to achieve successful UC systems. In this section we are going to explore the most relevant works related to the introduction of plasticity characteristics in Ubiquitous Computing user interfaces and, by extension, in Distributed Physical User Interfaces.

### 2.1. Physical User Interface Development

The intrinsic nature and requirements of Ubiquitous Computing systems make them rely on natural user interfaces (NUIs) to interact with the users [2,3,12]. A common way to build NUIs for UC systems is by implementing customized DPUIs [4,5,13,14].

Physical User Interfaces take advantage of the properties and capacities of physical objects to bridge the gap between the users and the state of a digital system [2,5]. Those objects range from everyday objects integrated in the environment to specific setups like dedicated appliances or even cockpits. These devices come from different manufacturers, use heterogeneous technologies, diverse modalities, and in some scenarios, even use custom hardware especially built for a particular system.

Home Automation and Internet of Things (IoT) technologies are a common example of technologies used to build PUIs for ubiquitous computing systems because they allow the transformation of typical objects found in homes and buildings into connected devices that can be controlled and accessed by UC applications. Home automation technologies [15,16] and IoT technologies [17,18] share a common pitfall, they are not interoperable at all. Consequently, one is limited to the devices available for each technology.

A recent approach to increase the interoperability of these solutions is the Apple HomeKit project [19]. It is a framework for iOS devices that allows the utilization, with the same API, of different distributed devices from a variety of manufacturers. HomeKit uses devices as accessories of iOS devices, allowing their control and the configuration of joint automated actions.

Even though home automation and IoT technologies are required to build Physical User Interfaces integrated in the environment, they have not been designed with that purpose in mind. Therefore, in order to foster the development of PUIs, the research community has introduced several PUI development frameworks that are worth mentioning.

Phidgets [20] is one of the first and most interesting PUI development frameworks. It includes a series of prebuilt physical devices that resemble typical graphical widgets, and a hardware development platform to build new physical widgets. On top of that, an API allows developers to access the diverse Phidgets functionalities. Phidgets are connected to a computer via USB, so they don’t support distributed UIs.

VoodooIO [21] follows a similar approach to Phidgets, but it is especially focused on gaming UIs. VoodooIO provides a kit of components (the VoodooIO Gaming Kit, VGK) that allows video-game players to build their own video game controllers using a set of atomic components and changing their layout. These custom controllers are then connected to a computer using USB and the standard driver APIs for keyboard, mouse or joysticks. With VoodooIO, players are able to build customized physical cockpits for each game, thus enhancing the experience of playing games like flight or driving simulators.

iStuff [6], which is part of the iROS and iRoom projects [22], is a framework for the development and prototyping of post-desktop ubiquitous computing user interfaces. iStuff components are wireless physical devices that are connected to a machine running a proxy software that encapsulates the device behavior and connects it to the iROS operating environment. iStuff uses the iROS infrastructure to allow distributed access to the component proxies and provides a virtual “patch-panel” that facilitates the mapping between devices and applications, thus making it quite easy for developers to try different configurations of a PUI.

EIToolkit [7] follows a very similar approach to iStuff. It also connects applications to devices by using a software platform that relies on proxies to encapsulate the protocols used by each device.

Even though those frameworks are closer to UI technologies than Home Automation and IoT technologies, a common drawback of these solutions is that they are more focused on device interconnection than on building user interfaces. As a consequence, their APIs and protocols are built on top of device concepts and not user interaction ones. Furthermore, they lack adequate device abstraction capabilities, thus requiring modifications in the code in order to integrate new devices.

From this review, we can conclude that there exists a need to increase the level of isolation between application developers and the devices or appliances that make up a distributed physical UI. A common and homogeneous user interaction API to access any kind of interaction resource, using any available technology and modality, will enable developers to build their DPUIs independently of the interaction resources used, allowing them to change the devices without affecting the application. Furthermore, it would allow the development of autonomous or semi-autonomous solutions to manage the change of IRs at runtime.

### 2.2. Plasticity in Physical User Interfaces

Ubiquitous Computing UIs commonly rely on a mix of traditional (graphical) UIs and a set of heterogeneous interaction resources (IRs) [4,5,6], which can range from physical devices embedded in the environment to custom designed devices, tangible UIs, or even voice or gesture recognition systems. As a result of this, unlike traditional software systems, where if the user moves to a new location the interaction resources hardly change, in UC systems a change of context may imply a dramatic change in the IRs available (the hardware platform) for the UI. Furthermore, not only the hardware platform could be different, but the new characteristics of the environment or the users can render the previous UI inadequate. Therefore, in UC systems, the adaptation of the UI to the new context, this is, the plasticity of their UIs, is essential to keep the system within the margins of usability and transparency required.

The Model-Driven Engineering (MDE) methodology and model-based techniques are the foundation of the majority of the existing approaches for building plastic UIs. This is not a coincidence, but one of the consequences of the characteristics of model-based techniques. With MDE, the knowledge about the system, the users and the environment is stored in machine readable models, allowing the system to exploit them, even at runtime, to modify and adapt its behavior according to the information provided by the models and the current context for each scenario.

Thevenin and Coutaz introduced the term plasticity of user interfaces in 1999 [11] and proposed a conceptual MDE framework to support the development of plastic UIs. This theoretical framework establishes general guidelines for techniques and tools to foster the development of plastic user interfaces. They propose the specification of the UI as a set of models providing abstract and declarative descriptions of the interaction capabilities of the UI and the physical environment where the UI will be executed. A series of tools, either automatic or semiautomatic, exploit those models by transforming them through a series of decreasing levels of abstraction until the implementation is achieved. The work of these authors led to the creation of the Unifying Reference Framework [23,24] inside de Cameleon project, providing a reference design and guideline for the development of plastic UIs using MDE techniques.

The Cameleon framework represents a major milestone in the development of MDE techniques for UI plasticity because, even though it is a theoretical framework, it has inspired and served as a foundation for many of further developments in the field of UI plasticity [25]. The Cameleon framework defines a set of initial models to describe the context of execution of a UI, and four additional models to specify the UI at four different levels of abstraction. Those additional models are supposed to be inferred after the initial models, either manually or automatically, and they represent abstract specifications (at different levels of abstraction) of the UI. It is important to highlight that, while Cameleon is a theoretical framework, it has fostered the emergence of many implementations following its principles.

A prominent example inspired by Cameleon is the USer Interface eXtensible Markup Language (USIXML) [26]. It is a model-based framework constructed around a user interface definition language (UIDL), the USIXML set of languages, which support the development of UIs using a multi-directional development method that allows developers to start the development from any, and even multiple, levels of abstraction, and proceed by transforming those models towards obtaining one or many final UIs.

Other examples are the Transformation Environment for inteRactivE Systems representAtions [27] (TERESA), and its continuation project, Model-based lAnguage foR Interactive Applications (MARIA) [28]. They start the development of UIs at the abstract level, with a single model specifying the tasks and different contexts supported by the application. Next, this abstract task model is transformed into a series of specific task models for each target platform, to then, generate an abstract UI model for each platform. In the last step, a platform dependent final UI for each platform is generated from each abstract UI.

Frameworks like TERESA, MARIA or UsiXML represent the most cited approaches to UI generation using MDE approaches. They are general purpose frameworks based on their own UI definition languages and populated by many different tools for a variety of specific purposes like web UI generation, graphical UI generation for different platforms, or voice UI generation. Nevertheless, they lack specific support for Ubiquitous Computing user interfaces, and more in particular Physical User Interfaces.

Model-based techniques have also been used to achieve UI plasticity in the context of ubiquitous computing. Two prominent examples are Dynamo-AID [29] and MASP [9].

Dynamo-AID is as model-based UI development framework for multiple devices. The core idea behind it is to rely on task models to drive the generation and adaptation of the UI to different services and devices. Dynamo-AID keeps tasks as the main concept of the framework, so the distribution and multimodal support is also related to tasks, supporting only the distribution of tasks and the utilization of different modalities for each task.

The Multi-Access Service Platform (MASP) follows the ideas of the Cameleon framework and USIXML, specifying the UI by employing a set of models at different levels of abstraction. The main contribution of MASP is that the transformation of models is done at run-time, with the models evolving in memory during the execution of the application. MASP transforms, at run-time, the components of the abstract model into concrete components that use three different modalities: WIMP web applications (windows, icons, menus, pointer), voice recognition, and gesture recognition. Furthermore, the different concrete components can be rendered in a variety of distributed devices like PCs, smartphones or tablets.

Out of the MDE area, ICrafter [30] and Personal Universal Controller (PUC) [10] are two of the main approaches to address the problem of UI development in Ubiquitous Computing environments. Both of those projects have taken a similar approach to UC user interfaces. They generate custom graphical UI (XHTML) or voice-based UIs (VoiceXML) to remotely control the appliances and devices available in a ubiquitous computing environment from a PC, a PDA or a smartphone.

All of these projects, ICrafter, PUC, Dynamo-AID and MASP follow a very similar approach. They generate graphical or voice UIs for different end devices, like smartphones or tablets, starting from some kind of models describing the UI or the capabilities of remote appliances. While those projects are focused on Ubiquitous Computing and Ambient Intelligence systems, and they support the generation of UIs adapted to each end device used for interaction, the fact is that they ignore the problem of physical user interfaces by relying mainly on remotely rendered GUIs and voice UIs.

Therefore, as can be seen from this review, we can conclude that there is a lack of support for the development of distributed physical user interfaces, where devices of very different natures and interaction capabilities are used to build ubiquitous user interfaces that provide a natural user experience in a particular environment. Even more importantly, there is a clear lack of solutions that are portable/adaptable to different scenarios.

The solution proposed in this paper is built on top of model-based techniques inspired by approaches like USIXML, Cameleon-RT, MASP or PUC. But compared to these previous works, it will make possible the development of AmI UIs capable of using any kind of IR, based on any hardware platform, and independently of its physical location in the environment.

## 3. A Framework for Developing Adaptable Ubiquitous User Interfaces

Ubiquitous Computing systems characteristics, objectives and constraints affect the nature of their User Interfaces (UIs), making them quite different from the UIs of classical software systems. First of all, UC systems are expected to operate in a proactive and intelligent manner, providing their functionalities while staying out of the way of the users and requiring minimal interaction with them. Second, this interaction is expected to happen in a natural way, adapted to the characteristics of the situation, the user preferences and abilities, the environment characteristics, and the interactive devices available in each location. For this reason, in Ubiquitous Computing, Windows, Icons, Menus, Pointer (WIMP) user interfaces are the exception, and Natural User Interfaces (NUIs) implemented with Physical User Interfaces (PUIs) are the norm [1,2,3,4,5,6,7]. Last, but not least, UC systems are expected to be ubiquitous or, at least, to seem ubiquitous, by providing their functionalities in many places.

If these three characteristics are combined, we can see how different UC UIs are compared to classical GUIs. They must not only be proactive and transparent, but they must also be adapted to the context, and, if that were not enough, they must be able to operate in different places, with different characteristics, disparate devices and multiple users.

A common naive technique to address these problems is to design and build a different UI for each combination of user, environment and use case. Nevertheless, as can be easily imagined, this solution does not scale well and, in many cases, requires a huge amount of effort. Furthermore, any scenario that has not been predicted at design time would be unsupported.

**Figure 1:**
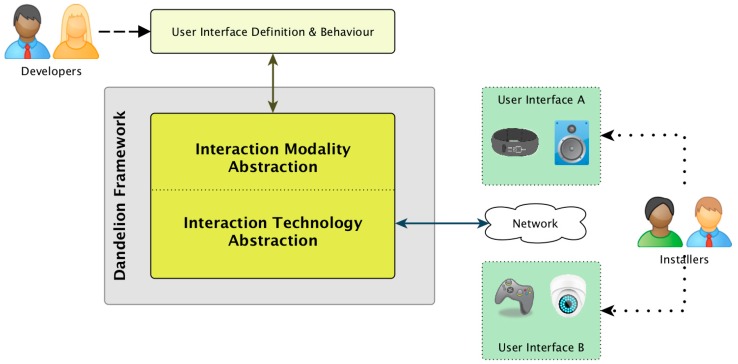
Overview of the Dandelion distributed architecture.

In this paper we propose to address these problems by increasing the level of decoupling between the system, the UI code and the interaction resources that build the UI. The core idea is to introduce a new framework for Ubiquitous Computing UI development that highly increases the decoupling between the business logic, the UI control logic and the interaction resources. This framework, called Dandelion, follows a MDE approach, using models to store and manage the information that will drive the adaptation of the UIs to the environment, and, as can be seen in Figure 1, revolves around two big levels of abstraction between the UI and the diverse interaction resources that build up a final Ubiquitous Computing UI.

The first level of abstraction, that we call Interaction Modality Abstraction (IMA) has the goal of logically decoupling the UI design and UI behavior logic from the specific interaction modalities and technologies of the interaction resources (hardware devices or software components) that build the final UI. The IMA isolates the developer’s knowledge from the particularities of each device, allowing developers to design UIs and write their application code without knowledge about interaction modalities or APIs used to render the UI for each usage scenario.

The second level of abstraction is the Interaction Technology Abstraction (ITA). It has the goal of isolating application and UI code from the particular set of devices used to interact with the user, including their APIs, protocols and their physical location. The ITA allows developers to build distributed UIs without extra effort or knowledge about network protocols, APIs, etc. Furthermore, the ITA also allows installers to physically deploy the UI with different sets of interaction devices without requiring any modification to the application or UI control code. To achieve this, the ITA relies on a repository of Dandelion compatible devices. This repository is a key aspect of the solution, because, as will be seen in the Section 4.2, only if there are support for many, and in particular, the necessary devices for a UI, the development costs and complexity can be effectively reduced.

The combination of these two layers, IMA + ITA, can make the development of systems capable of true ubiquitous interaction easier. They facilitate and reduce the costs of developing UIs capable of adapting to changing scenarios, using multiple modalities to interact with the user and distributed devices to provide ubiquity support. The remainder of this section is devoted to exploring the Dandelion framework and how it realizes this two abstraction layers into a distributed UI development framework.

### 3.1. Overview of the Dandelion Framework Architecture

**Figure 2:**
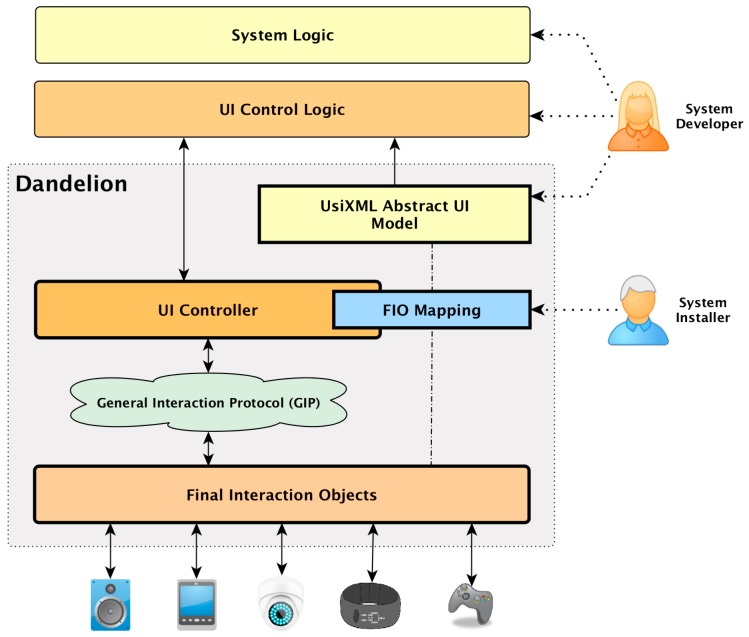
Dandelion detailed architecture block diagram.

Figure 2 shows the software architecture of the Dandelion framework. As can be seen, by using Dandelion, UC developers are decoupled from the specific modalities, technologies and even physical location of the Interaction Resources (IRs) used to implement a particular DPUI. Developers can design and describe the UIs at the abstract level using the UsiXML language, and then implement the application UI control logic on top of the abstract concepts defined in the abstract UI. Dandelion then uses a distributed user interaction controller to connect those abstract elements to the physical elements that perform the interaction with the user. This connection is managed by Dandelion itself, who performs the translation from the abstract concepts to the real interaction with the user. It does so by relying on a series of distributed proxy-like components that elevate any kind of device or software component to the status of an Interaction Resource (IR). They provide a common interface of interaction operations that is remotely accessible through a networking protocol called the Generic Interaction Protocol (GIP) [31].

Following the different components shown in Figure 2, it can be seen how the two different abstraction levels proposed, IMA and ITA, are realized by Dandelion in order to decouple developers from the final shape of the UI for each scenario.

The IMA level is provided by the utilization of model-driven engineering techniques to design UIs at a declarative and abstract level using the UsiXML Abstract UI model, and then implement the UI control logic on top of those abstract concepts. Therefore, as can be seen in Figure 1 and Figure 2, the UI designer and the UI control logic are effectively decoupled from the concrete interaction resources used, that must only know by the system installer.

The IMA is the foundation of the Dandelion framework. It provides the most basic and important abstraction feature, decoupling the UI control logic and the UI design from the IRs’ specific modalities and APIs. From the developer’s point of view, it provides a common conceptual model to support the design and implementation of the UI control logic of UC applications. This model, the Abstract Interaction Model (a more detailed description is provided in Section 3.3.1) must provide conceptual abstractions for all the common interaction concepts, so that developers have enough freedom to support the majority of interaction scenarios. Developers design and build the UI control logic on top of those abstract concepts, instead of specific elements from concrete technologies, and let the IMA provide translations between those abstracted interaction concepts and the specific APIs of the interaction resources used for each scenario. This translation is performed by the ITA implementation, which physically connects the abstract interaction elements managed by the developers with the physical interaction resources that realize the UI for a specific scenario. More details about how Dandelion implements the IMA and decouples developers from interaction modalities is provided in Section 3.4 and Section 3.5.

The Interaction Technology Abstraction (ITA) implementation of Dandelion transforms any UI implemented on top of the IMA into a distributed UI, thus decoupling the application code from the concrete set of physical devices that build up a particular UI. Dandelion achieves this decoupling by physically separating the abstract elements managed by the UI code from the interaction devices that are physically deployed in each environment. For this purpose Dandelion provides an implementation of the Generic Interaction Protocol [8], a distributed interaction protocol designed to mimic the conceptual interaction operations supported by the Abstract Interaction Model. Dandelion translates the interaction actions performed by the UI logic on top of generic interaction elements into events in the GIP protocol that are sent, over the network, to the remote devices that will implement the real interaction with the user. Any element that implements the GIP protocol is called a Final Interaction Object (FIO), and they are in charge of translating abstract interaction actions into real and physical interactions with a user using a concrete device. With the introduction of the Generic Interaction Protocol, for which a more detailed description is provided in Section 3.5.1, the UI logic is now not only decoupled from the specific technologies and APIs of each IR, but it is also decoupled from their physical location. This opens the possibility of building ubiquitous UIs that can be implemented using multiple IRs, with different modalities and deployed in different physical places. More details of the ITA are provided in Section 3.5.

Finally, the IMA + ITA combination, which allows the design and implementation of distributed physical UIs without specific knowledge of the underlying IR technologies, is provided by managing a dynamic mapping between the abstract interaction elements of the UI and a specific set of FIOs for each usage scenario.

As a final note about the Dandelion framework, it is important to mention that the implementation of the framework described here has been released as open source software under the GNU Affero GPL v3 license, and it is accessible through a public GitHub repository [32].

### 3.2. The Dandelion UI Development Process

**Figure 3:**
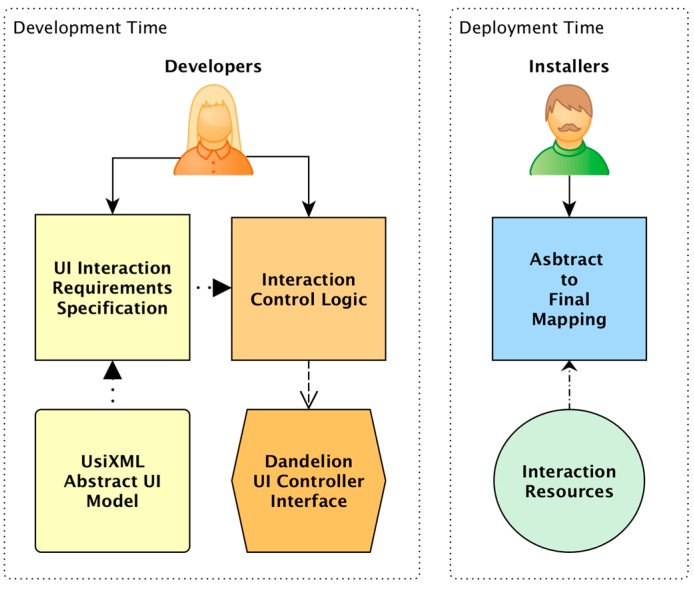
Dandelion adaptable UIs development process.

Before going into an in-depth description of the Dandelion implementation, in this section we are going to provide a brief description of the development process that developers must follow in order to build UIs using Dandelion. This process is strongly influenced by the use of Model-Driven Engineering techniques and by the three different aspects in which Dandelion improves the decoupling between the developers and the UI. This reduces the design and development of Ubiquitous Computing UIs to a three step procedure as shown in Figure 3.

The first step is to design the user interface. For that purpose, Dandelion provides an implementation of the IMA Abstract Interaction Model, so that developers can describe their UIs using generic interaction elements, thus working at a high level of abstraction between them and the interaction resources. The second step is to write the user interface behavior logic. Dandelion provides developers with a generic interaction API, inspired by this Abstract Interaction Model, which allows them to implement the UI behavior logic using generic interaction operations. The final step is to connect this generic definition of the UI to the real devices that will implement the UI and, thus, interact with the user. Dandelion itself is in charge of performing the translation between the generic interaction elements, and the generic interaction, into real operations over real and physical interaction resources. Nevertheless, without an implementation of the ICA, it requires developers or installers to manually specify which devices will implement each interaction element.

This process of building a UI makes Dandelion quite different from the majority of MDE development frameworks. In Dandelion only the first two steps are performed during development time, and they do not require knowledge about specific technologies, devices or usage scenarios. The third and last step is the only one that requires specific knowledge of devices and usage scenarios, but it is performed at deployment time, thus it is isolated from the development and, therefore, does not require developers to modify the application at all.

MDE frameworks for UI development usually employ models as a supportive tool to guide developers during the development of the system. Thus, the transformation from abstract to final is carried out by the developers at development time. Consequently, the set-up of the scenario (the IRs available and the characteristics of the environment) is not known. For this reason, these frameworks start from the abstract UI and produce different versions of the UI at different levels of abstraction. First, the concrete UI level, where the interaction modalities are already selected, and then, the final UIs, which are multiple versions of each modality where the final implementation technologies or APIs are decided.

Unlike these systems, Dandelion performs the transformation at run-time, when the characteristics of the scenario are already known. Therefore, we can avoid the intermediate transformation steps and go directly from the abstract to the final UI because we know which IRs are available, which are the characteristics of the environment and the user, etc. We don’t have to start by selecting the modality and then the concrete implementation. We can already start by selecting particular IRs to implement each of the interaction actions specified in the abstract UI model.

By taking advantage of this characteristic of Dandelion, developers can work completely at the abstract level following the process illustrated in Figure 3. It is at deployment time where the transformation from abstract to final is specified by the installer of the system. This specification, essentially a mapping between abstract and final components, is used by Dandelion at run-time to translate the abstract interaction operations into real interactions with the users.

In the following subsections we are going to explore in detail how the Dandelion Framework supports these three steps, and how developers can use them to build portable user interfaces for Ubiquitous Computing systems more easily.

### 3.3. Decoupling User Interface Design from Interaction Modalities

As previously introduced, in Dandelion, like almost any UI development framework, the first step for developing portable Physical UIs is to design the UI. The key characteristic of Dandelion is that this design must be performed at a very abstract and conceptual level. Developers are only required to specify the user interaction requirements of the application, which can be done using the Abstract UI model. This model provides support for a reduced set of only five different abstract user interaction primitives:

- The user can input information to the system.
- The system can output information to the user.
- The user can select information from a collection shown by the system.
- The user can request the execution of an action by system.
- The system can request the focus of the user to a specific element of the UI.

Consequently, the user interaction requirements specification of an application is reduced to answering the next questions:

- How many information elements are going to be shown to the user?
- How many information elements are going to be introduced by the user?
- What type of information is going to be shown or introduced?
- How many interaction actions is the user allowed to perform?
- How the different components are organized and related to each other in the UI?

This specification of the UI requirements is achieved using the Dandelion Abstract UI model, which is inspired by the UsiXML Abstract UI Model (AUIm). As indicated in the next subsection, this model introduces the Abstract Interaction Unit concept (AIU) as an abstract representation of the widgets found in classical GUI toolkits. The AIUs work as containers for interaction primitives, thus allowing the specification of the relations between the different user interaction elements. Furthermore, AIUs can also contain other AIUs, thus permitting the specification of the UI shape at an abstract level using a hierarchical organization.

#### 3.3.1. The Abstract Interaction Model

The Abstract Interaction Model is the main medium of interaction between developers, UI code, and the framework. Its purpose is twofold. First, it must provide developers with a common model to describe the interaction requirements of their UIs. Second, it must provide a common set of operations to make use of the available Interaction Resources.

In order to accomplish the first goal, the Abstract Interaction Model should be able to represent very different Interaction Resources, modalities, and technologies behind a reduced set of generic concepts. But, in order to achieve the second goal, this reduced set of concepts should provide developers with enough expressive power to describe the user interaction requirements of almost any UI.

With so many authors working on the design and development of models to describe UIs at different levels of abstraction, especially following the abstraction level division of Thevenin and Coutaz [24], we decided that the best way to go would be to rely on one of those solutions for the definition of the Abstract Interaction Model of the TIAF. As shown in Section 2.2, three prominent approaches are the TERESA project [27], the MARIA project [28] and the UsiXML project [26]. Inspired by the ideas of the Cameleon conceptual framework, each of those projects have been building a complete model-based UI development framework. At the core of those frameworks, there are a set of models that allow developers to describe the different aspects of an UI at different levels of abstraction.

The most interesting models for our purposes are the Abstract Interface Models. In all of the cited frameworks, they serve the same purpose as in Dandelion; they provide developers with a generic set of concepts, independent of any technology or modality, to describe the interaction requirements of the UIs. With this in mind, we decided to use the Abstract User Interface Model of the UsiXML [26] project as direct inspiration for the Abstract Interaction Model of Dandelion. It has great expressive power, a lot of tools to create and manage models, and more importantly, this conceptual model has been proposed by the W3C for the definition of a standard Interface Definition Language (IDL) for abstract user interfaces [33].

**Figure 4:**
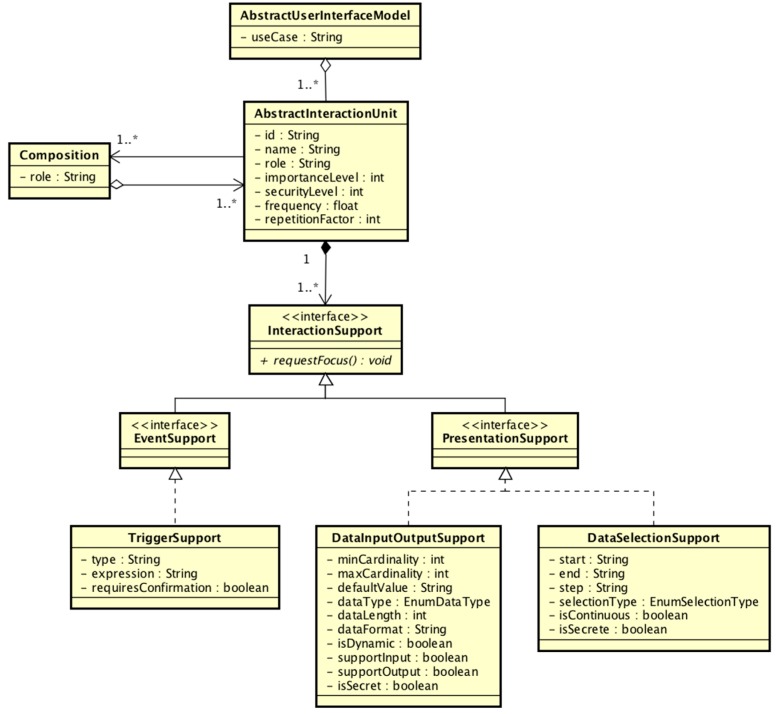
Class conceptual model of the Abstract Interaction Model directly inspired by the UsiXML Abstract User Interface Model.

As shown in Figure 4, the main concept of the AUI Model is the Abstract Interaction Unit (AIU). It is a generic representation of the typical widgets found in graphical user interface toolkits. Each AIU is associated to a set of different interaction facets (input, output, selection, etc.) that represent the interaction capabilities required by the AIU. Furthermore, AIUs can be organized into hierarchies by defining AIUs that are composed of other AIUs.

While the AIU is the main organizing element of the model, the interaction facets, InteractionSupport, EventSupport, and PresentationSupport, are the elements that provide expressive power to it. By combining the three concepts available in the model, it is possible to describe an AIU that requires five different interaction actions:

- *Action.* The TriggerSupport indicates that the AIU requires some kind of support to trigger actions. Like a button in a GUI.
- *Input.* DataInputOutputSupport can be used to indicate that the AIU requires support to input, output, or both, some kind of data from the user.
- *Output*. The same as above.
- *Selection*. The DataSelectionSupport indicates the requirement to support a selection of an item among a collection of them.
- *Focus.* All the interaction support elements share a common interaction operation to request the attention of the user.

In order to describe a complete user interface, developers only have to define a collection of interrelated AIUs, with each AIU describing a set of user interaction operations requirements. As can be seen, the UsiXML AIU Model defines quite a reduced set of generic user interaction concepts, but it provides a great amount of expressive power, enough to describe even large and complex interfaces, with multiple interaction requirements [25].

#### 3.3.2. Illustration Example

In order to facilitate the comprehension of how the different concepts provided by the Abstract UI Model can be used to design UIs from an abstract point of view, we are going to explore an example of abstract UI design and specification in the rest of this subsection.

Let us imagine a ubiquitous music player that is able to play music wherever the user is. The player, that we have called the Environmental Music Player (EMP) must not only play music in many different locations and situations, but also must provide the user with adequate mechanisms to interact with the EMP and stop the music, change the song or modify the volume level.

**Figure 5:**
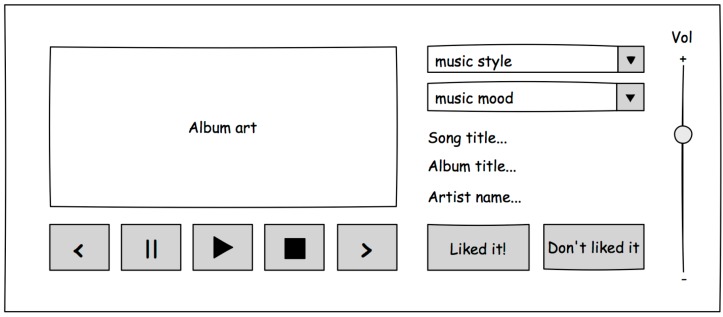
Sketched UI of the Environmental Music Player using WIMP user interfaces.

Figure 5 shows a sketched version of a classical graphical user interface for the EMP application. As it can be seen, it has all the typical controls of a music player. It lacks a song selection UI because, in order to keep the example simple, we have designed it as an autonomous player that manages a collection of music and plays songs of a particular music style or mood.

So, the first step with Dandelion will be to specify the user interaction requirements of the graphical UI shown on Figure 5. Algorithm 1 shows a reduced version of the specification of the player controls part of the EMP UI. This part of the UI is in charge of providing the user with the required controls to manage the playing of music. This includes the ability to change from one song to the next, start or stop playing music, change the music style, or change the volume of the audio.


**Algorithm 1.** Reduced XML code describing the Abstract UI model for the EMP.*...**<aui:AbstractInteractionUnit id="PlayerControl"">*
  *<aui:Composition rationale="player control panel">*    *<aui:AbstractInteractionUnit id="PlayStop">*      *<aui:TriggerFacet id="PlayAction">*        *<aui:triggerType>operation</aui:triggerType>*      *</aui:TriggerFacet>*      *<aui:TriggerFacet id="StopAction">*        *<aui:triggerType>operation</aui:triggerType>*      *</aui:TriggerFacet>*    *</aui:AbstractInteractionUnit>*    *...*    *<aui:AbstractInteractionUnit id="MusicStyleControl">*      *<aui:DataSelectionFacet id="MusicStyleSelector"*      *isContinuous="false" selectionType="SINGLE">*        *<aui:dataType>text</aui:dataType>*    *</aui:DataSelectionFacet>*    *…*  *</aui:Composition>**</aui:AbstractInteractionUnit>**...**<aui:AbstractInteractionUnit id="MusicMetadata">*  *<aui:Composition rationale="player music metadata panel">*    *...*    *<aui:AbstractInteractionUnit id="AlbumMetadata">*      *<aui:DataInputOutputSupport id="AlbumCover"*      *dataFormat="image" inputSupport="false" outputSupport="true">*        *<aui:dataType>image</aui:dataType>*      *</aui:DataInputOutputSupport>*      *<aui:DataInputOutputSupport id="AlbumTitle"*      *dataFormat="string" inputSupport="false" outputSupport="true">*        *<aui:dataType>text</aui:dataType>*      *</aui:DataInputOutputSupport> ...*    *</aui:AbstractInteractionUnit>*    *...*  *</aui:Composition>**</aui:AbstractInteractionUnit>**…*


Regarding the muysic style selection, it is not easy to model it as individual actions, because the number of music styles available can be dynamic and vary depending on the collection of music available. Therefore, it is mandatory to model it as a selection primitive using text items. These items will be dynamically provided by the application logic at runtime.

The example of the EMP UI is also useful to show how the Abstract Interaction Unit concept can be used in different ways to organize the UI elements. In the control part of the UI, we use a main AIU as container for all the controls (imagine it as a kind of an internal panel in a GUI), and then, we use three AIUs to organize the different facets required by the UI. One AIU for music player controls, one for song selection, and one for volume selection. This organizational information is not only useful to facilitate the design by taking advantage of the famous divide and conquer strategy, but it can also be used by Dandelion as hints to select and manage the final and physical realization of the UI. For example, keeping the controls together, physically separating controls of different AIUs, etc.

It is important to note that input and output are not relegated only to basic data types, like numbers or text. They support a set of data types including images. For example, in the case of the EMP, it requests to output the images of the album art for each song. In the code listing of Algorithm 1, we display how a DataInputOutputFacet can be used to output/input images to/from the user.

### 3.4. Decoupling User Interface Logic from Device’s APIs

Recalling the process shown in Figure 3, once the UI has been designed and specified at the abstract level, the next step is to implement the UI logic code. This logic is specific for each application and it is in charge of defining how it uses the different UI elements in order to exchange information between the system and the user. It specifies, for example, when a message is going to be shown to the user, what to do with the information introduced by the user, or what business logic action to execute when the user issues a specific UI action. More generally speaking, the job of the UI control logic is to fill the gap between the business logic and the UI, thus decoupling one from the other.

In Dandelion, given that the UI is designed at the abstract level, the UI control logic can also be implemented on top of the same abstract UI concepts. It provides developers with an external façade, the Dandelion UI Controller (DUIC), that exposes the functionality of the framework through a reduced set of operations tightly related to the abstract concepts proposed by the Abstract UI Model. All the operations exported by the DUIC interface are executed over abstract elements (AIUs and interaction facets), which are then translated by the Dandelion User Interface Controller into real operations over the IRs. Thanks to that, by building the interaction control logic on top of this set of abstract interaction operations, developers are able to completely decouple that logic from the technologies, APIs, and specific hardware of the Interaction Resources (IRs).

The reduced set of operations of the DUIC supports all the five interaction primitives proposed by the Abstract UI Model. In particular, it allows the system to request Dandelion to:

- Show some information to the user.
- Show a collection of information items to the user, and let she select one of them.
- Gain the user focus over a specific element (AIU) of the UI.
- Notify a callback system object when the user inputs some information.
- Notify a callback system object when the user triggers the execution of some action.

It is noteworthy that all of these operations are abstract, and neither the UI model nor the UI control logic specify how they are implemented. As we will see in more in the following subsections, it is the responsibility of the DUIC to provide a concrete implementation of those abstract operations, specifying, for example, how the UI is going to gain the focus of the user over a specific part of it, or how the UI is going to show an information message to the user.

#### Illustration Example

Just to clarify how the DUIC interface can be used to implement the UI control logic, let’s examine some small code examples from the EMP UI example. Algorithm 2 shows a small JAVA code snippet from the EMP example. This code uses the DUIC façade to configure the callback objects from the business logic, that must be called by Dandelion when the user triggers the actions “PlayAction” or “StopAction” specified in the abstract UI.
**Algorithm 2.** Control Logic code required to set the business logic actions that must be called when the user triggers the actions to start playing music in the EMP example.*//the player controls “panel”**AbstractInteractionUnit playerControlAIU =*   *app.getAbstractUI().getAbstractInteractionUnitById("PlayStop")*;
*//the play “button”**TriggerFacet playTriggerFacet = (TriggerFacet) playerControlAIU.getInteractionFacetById("PlayAction");*
*//register the action to be called when the “play” action is activated by the user**dandelionUIC.registerActionCallback(*   *playerControlAIU, playTriggerFacet, new PlayActionCallback(musicPlayer))*;



Continuing with the example of the EMP, the code listed in Algorithm 3 shows how to use the DUIC interface to output a string message to the user, in this case the title of the song that the EMP is playing. As can be seen in these small examples, the implementation of the UI Control Logic is completely free of any details about how the UI is going to be finally implemented. It is coded just on top of the abstract interaction elements specified in the Abstract UI Model and by using abstract interaction operations and primitives.
**Algorithm 3.** UI Control Logic code required to output the song title string to the user. It is executed each time the song changes.*//show the song title to the user**dandelionUIC.showOutput(*   *currentSong.getID3v1Tag().getSongTitle(), songMetadataAIU*,   *songTitleFacet, new Hash- Set<FuzzyVariable>(0))*;



Finally, it is important to note that, unfortunately, the fact of designing the UI and implementing the control logic at such a high level of abstraction has an important drawback. As happens with any kind of abstraction technology, many details and particular capabilities of the IRs are hidden to the developers, thus hindering the fine grain customization of the UI and the user experience. It is more difficult for developers to take advantage of all the particular possibilities of each technology and device, and it is more difficult to implement highly customized UI behaviors. This is a common and, to some extent unavoidable, issue of abstraction technologies. However, in Dandelion each abstract interaction operation supported by the DUIC can be customized by a collection of Interaction Hints (IHs). They are fuzzy variables indicating suggestions or hints to the DUIC about how the developers want that operation to be implemented. Some examples of Interaction Hints are the privacy level, the importance level or the color used to show an interaction operation.

### 3.5. Decoupling User Interface from Device’s Deployment

As shown in Figure 3, once the UI has been designed and the UI control logic implemented, the system is ready for deployment. It is at that point where the final UI has to be assembled for each specific usage scenario.

**Figure 6:**
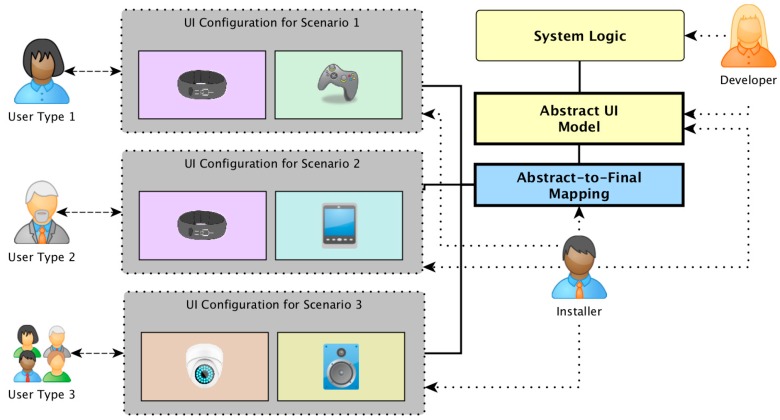
Many different UIs can be deployed for the same Abstract UI definition and the same UI Control Logic. The installer of the system only has to specify different mappings for each scenario.

As shown in Figure 6, the selection of the IRs to build the final UI is the only point where the developers or installers are not decoupled from the context and the particular devices available. This is because they must bear in mind the characteristics of the context (environment, user, use case, etc.) and the characteristics of the IRs (modalities, physical characteristics, etc.), but this happens only at deploy time.

The IMA and ITA layers are highly related because the physical decoupling between UI logic and the devices is directly implemented using the GIP and remote proxy components that work as abstractions of the real IRs available. As the GIP continues to provide an abstract interface of interaction primitives directly inspired by the UsiXML Abstract UI model, the process of building the final UI consists exclusively on selecting, among the physical IRs available, those that better match the needs of the scenario, and then, establishing a mapping between the different interaction facets specified in the UI abstract model and the interaction capabilities of the IRs.

The ITA implementation in Dandelion is built on top of three main software components (see Figure 2):

- The UI Controller. It is the link between the IMA and the ITA layers.
- The General Interaction Protocol (GIP). It provides distributed access to the IRs using an interface of generic user interaction operations inspired by the Abstract UI model.
- The Final Interaction Objects (FIOs). They provide concrete implementations of the GIP interface, translating the generic abstract concepts and operations to the particular APIs of each IR supported.

The UI Controller lies between the IMA and ITA layer. It is an implementation of the Interaction Realization Module of the IMA but, thanks to the GIP, its behavior is quite simple, it operates as a router of abstract user interaction operations.

#### 3.5.1. The Generic Interaction Protocol

The Generic Interaction Protocol [8] is used by Dandelion to provide the decoupling between the system and the IRs at two levels. First, it is the main element that encapsulates the behavior of heterogeneous IRs behind a common set of abstract interaction operations. Second, as a distributed network protocol, it decouples the system from the physical location of the IRs. Because of that, even if it is more related to the ITA, in Dandelion the GIP is a key element of the IMA + ITA combination.

The Generic Interaction Protocol (GIP) [8] is conceptually conceived as a distributed communications protocol that creates a generic remote interface to any kind of interaction device. By implementing the GIP interface, any device or interaction resource can be accessed using the same set of concepts and operations, thus decoupling the application from the underlying interaction technologies and the location of the devices.

As can be seen in Figure 2, the GIP is right between the control logic and the interaction resources (physical devices, graphical widgets, voice recognition software, etc.), decoupling them in two ways. On the one hand, as the GIP is a distributed protocol, it provides physical decoupling between the system logic and the UI components. On the other hand, it provides logical decoupling by isolating interaction resources behind a common generic interface.

The GIP, combined with the UI Controller, provides a way to directly map Abstract User Interface (AUI) elements to the Final User Interface (FUI) elements at runtime. Since the actual IRs available are already known at the time the mapping is defined (run-time or deploy-time), the GIP eliminates the need for the definition of different CUI and FUI models for each modality and technology, because Dandelion allows developers to build the UI, and its control logic, at the abstract level and then connect that abstract logic, at run-time, directly to the final IRs available.

The common generic interface provided by the GIP is in charge of abstracting the behavior of concrete interaction resources. Thus, it should be generic enough to support multiple kinds of modalities and interaction devices, furthermore it must support all the expressive power of the Abstract Interaction Model. Therefore, the operations supported by the GIP are directly inspired by the Abstract Interaction Model and, because of that, by the UsiXML Abstract User Interface Model.

The GIP interface is designed to match the set of generic interaction facets described by the Abstract Interaction Model. Each IR implementing the GIP interface provides support for one or more interaction facets, thus facilitating establishing a mapping between an AIU interaction facet and an interaction resource that will perform that interaction.

Using the Dandelion framework, every AIU described in the abstract UI model of an application will be associated to a collection of IRs, each one of them implementing one interaction facet. Therefore, a complete UI will be a collection of physically distributed IRs, organized into AIUs and accessed through a remote interface of generic interaction operations.

In order to match the design of the Abstract Interaction Model, the GIP interface is made up of five different events: input, output, selection, action and focus. The first four events are directly inspired by the UsiXML AUI model:

- *Input*. An ir informs its subscribers that the user has performed a data input action.
- *Output*. The system logic commands one or many irs to output some data to the user.
- *Selection*. This event has two different meanings depending on the sender. If it is sent by an interaction resource, it means that the user has made a selection. Otherwise, it means that the system logic requires an interaction resource to show a selection to the user.
- *Action*. An interaction resource informs its subscribers that the user has triggered an action.
- *Focus*. The system logic requires an interaction resource to gain focus over the user attention.

It is not mandatory for every interaction resource to support all GIP events. There can be many kinds of interaction resources with different levels of support for user actions. Some will support input and output, while others will support only input or will not be able to reclaim the focus of the user.

As indicated in the previous section, in order to allow some level of customization of the user interface, GIP events are enhanced with a set of properties called Interaction Hints (IH). They are a set of fixed properties that developers can use to provide indications to the interaction resources about an interaction action (for example: priority, size or color). The support for IHs is not mandatory, and each interaction resource can interpret them as it wants.

#### 3.5.2. The Final Interaction Objects

The Final Interaction Objects are the end elements in charge of physically interacting with the user. They are software abstractions of heterogeneous interaction resources that could be either hardware (keyboards, remote controllers, appliances, sensors, etc.) or software (GUIs, voice recognition, etc.).

FIOs are implemented as software applications that implement the GIP remote interface. They can be implemented in any programming language, for any software platform, and using any API required by an IR. The only requirement to be compatible with Dandelion is to implement the GIP interface according to the JSON GIP codec of Dandelion, and to use a STOMP client to connect the FIO to the messaging broker. In fact, if the ActiveMQ broker is used, as suggested, it is even possible to use messaging protocols different from STOMP, because the broker handles the translation between protocols transparently.

As previously indicated, the FIOs assume the role of GIP publishers, abstracting the behavior of an IR as a set of events that notify the different actions the user is performing. But each particular FIO implementation is only required to support a subset of GIP events, as there can be IRs supporting only input, output, action, etc. The type and number of interaction facets supported by each FIO is defined in its description, which includes information like the type of data the FIO is able to manage, the cardinality of that data, the type of interaction supported, and the interaction modality used by the FIO. This is, each FIO describes its supported interaction facets, and it indicates the kind of data it can obtain from the user as input or selection, or show to the user as output. This data can be of any basic type: integer, float, string or boolean, and images in PNG format. It is important to note that each FIO can provide one or many different interaction facets. For example, a FIO can specify that it is able to output a string, input an integer, and receive an action from the user.

The FIO descriptions are used during the definition of the mapping from abstract to final in order to match the abstract interaction facets to a set of adequate FIO interaction facets. For example, the output interaction facet of an AIU for a string type can be associated to an LCD display encapsulated by a FIO with an output interaction facet supporting strings. To facilitate the management of the system and to make the autonomous selection of the FIOs in the future possible, Dandelion uses a FIO repository to store the descriptions of all the FIOs available. For that purpose, every FIO must register itself, at startup, in the FIO repository. The FIO repository is also connected to the Dandelion system using the messaging broker and a protocol using JSON and STOMP.

For each different kind of device or interaction software used by a system, a FIO must exist that abstracts it using the GIP. Obviously, a key point to facilitate the use of Dandelion is that developers should not need to develop their FIOs, or at least, not many of them. They should be provided by Dandelion itself or by the manufacturers of the interaction resources.

#### 3.5.3. Illustration Example

For illustrating how the same interaction can be realized and deployed using different interaction resources, in this subsection we are going to explore the particular implementation of the EMP music style selection action with different kinds of modalities and devices.

The Environmental Music Player does not play particular songs or albums. Instead, it allows the user to select a specific music style, and then it creates a playlist with all the songs, available in the user library, that match the selected style.

As we previously mentioned in Section 3.3.2, the music styles available for selection are dynamic and dependent on the collection of the user. Because of that, the selection of music styles must be modeled using a selection interaction primitive dynamically populated from the UI control logic. The code snippet presented in Algorithm 1 shows the definition of the selection interaction facet required for music style selection.

Once the abstract UI is defined, we must implement the UI control logic required to show the list of music styles to the user, and register a callback to receive the style selected by the user when she changes the selection. This code is implemented using the DUIC, without any knowledge of the particular devices.

**Figure 7:**
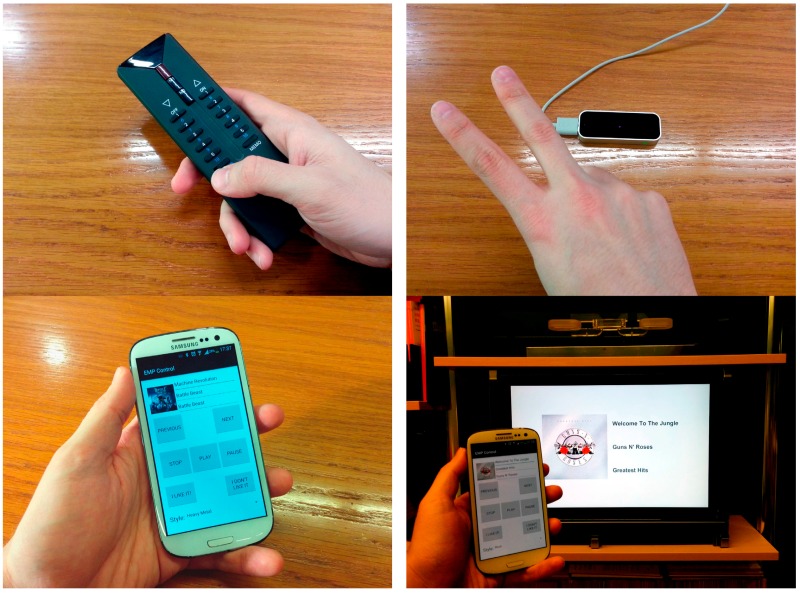
Four of the FIOs used for the selection of music style in the EMP example. First, a KNX home automation remote controller using buttons to select the style. Second, a Leap Motion device for finger and hand gesture recognition. Third, a custom Android GUI application. Fourth, a TV display for output with a Kinect camera for input of the style with gestures.

Finally, the music style selection interaction facet can be physically implemented in multiple ways using different FIOs to build various final UIs adapted to diverse usage scenarios. Figure 7 shows four different ways of physically implementing the selection of music styles: using a home automation device, in particular a KNX remote controller for home automation systems, hand and finger gestures to select between the different music styles using a Leap Motion device, as an Android application for smartphones that allows the selection of the music style using the touch screen of a smartphone and using a TV display and a Kinect camera.

In the case of the smartphone, it exports a simple GIP interface supporting only two interaction facets, selection and focus. This kind of selection FIO can be useful in many situations, it can be used to control de music style on-the-go, using a personal device, but it can also be useful at home. In the case of the TV display, it is used to show EMP information like artist, song title and album art, and input is supported by A Kinect camera to detect body gestures in order to select the music style.

All of these FIOs are implemented using the specific APIs of each device, and by implement the translation between the GIP interaction interface and the particular actions of each technology API.

## 4. Discussion

If something can be extracted from the literature review presented at the beginning of this paper, is that the field of Ubiquitous Computing user interface development is quite broad and still open. While there are solutions proposed to facilitate the development of this kind of UIs, the fact is that the majority are being designed and implemented ad-hoc for each particular system and scenario. This is because, even though many of the solutions proposed shine in some particular aspects, none of them seems to cover all of the aspects required to build plastic Distributed User Interfaces for Ubiquitous Computing systems.

In this section we provide a comparison between Dandelion and its main existing alternatives, so that the benefits of an integrated solution can be highlighted. We have performed this comparison from two main points of views. First, qualitatively, by establishing a feature comparison framework that allows us to compare the degree of support of different desirable characteristics of the available solutions. And second, quantitatively, by using objective metrics like lines of code or number of APIs required to implement a system.

### 4.1. Qualitative Analysis

In this subsection we compare the features of Dandelion with those of the existing solutions for the development UC UIs. The core idea is to compare them from a qualitative point of view, comparing the different degrees of support for a set of features that, from our experience with UC systems, are desirable for a UC UI development solutions. Starting from the review of the state of the art presented in Section 2 we have designed a comparison framework that includes ten different characteristics organized into three aspects:

- *User Interface Adaptation.* This group of features are about the capacity of a solution to adapt a particular UI to different contexts of use.
  ο *When*. At what point of the development is the adaptation of the UI supported. At design time? At development time? At Deployment time?
  ο *Type.* How is the adaptation performed? Automatically? Manually? Or not supported at all?
- *User Interface Development*. This group covers desirable characteristics of any framework for UI development.
  ο *Distribution of the user interface*. Degree of UI elements distribution supported by a solution. None: UI distribution not supported; Low: only the complete UI; Medium: only parts of the UI; High: all the elements of the UI can be distributed.
  ο *UI-centric development framework*. Whether a solution is based on user interaction concepts, or not.
  ο *UI abstraction*. The degree of decoupling between the UI developers/code and the particular technologies, modalities, and APIs used to implement the user interface. None: no abstraction at all; Low: Minor abstraction from devices; Medium: Abstraction from devices and technologies; High: Abstraction from modalities, technologies and devices.
  ο *Autonomous generation of the user interface*. Whether a solution supports the autonomous generation of user interfaces adapted to a particular usage scenario.
  ο *Capability to customize the generated user interface*. The degree of personalization of the final user interface produced with a particular solution. Low: Customization must be performed at development-time; Medium: Customization at deployment-time; High: Customization at runtime.
- *Physical User Interfaces*. This aspect includes important characteristics for the development of physical user interfaces.
  ο *Number of different modalities supported*. An indication of the number of different types of interaction modalities supported by a solution. Low: 1–3 modalities; Medium: 4–6 modalities; High: more that 6 modalities.
  ο *Number of different physical devices supported*. An indication of the number of different types of physical devices (used as interaction resources) supported. Low: 1–3 types of devices; Medium: 4–6 types of devices; High: more that 6 types of devices.

**Figure 8:**
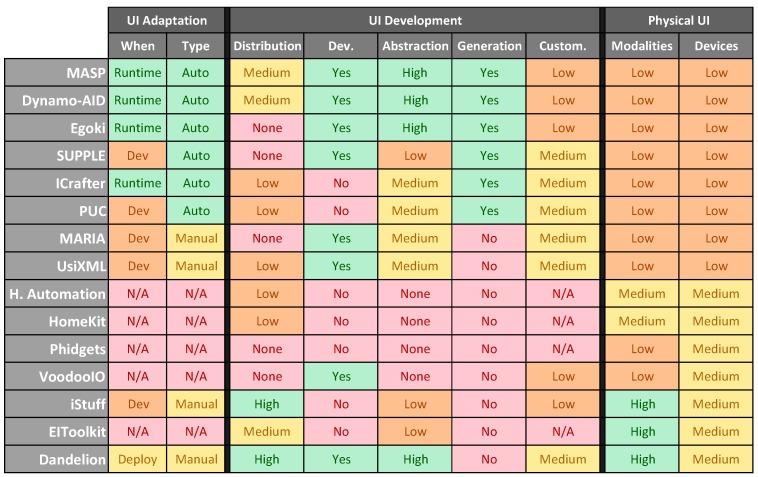
Feature comparison between the different solutions analyzed.

The results shown in table of Figure 8 are a clear reflection of the different varieties of solutions existing in the field. It can be seen how some groups of solutions perform really well in a set of aspects, but very poorly in others, and vice versa.

For example, there is a group of solutions that perform quite well in the Physical UI aspect, but they have really poor performance in UI Adaptation and UI Development features. This is the case of technologies like HomeKit, Phidgets, EIToolkit. While they can be used as supportive technologies to build ad-hoc PUIs, they are designed to provide remote access to physical devices, thus they are good at managing devices, but they do not support user interaction concepts, nor do they provide tools to design or build UIs.

Looking at the table it is also easy to see another big group of solutions. The first eight technologies displayed in the table have in common a very poor support for physical user interfaces. They support very few modalities (usually graphical and voice), and they do not include any kind of support for using physical devices as interaction resources. These technologies come from the user interface field, thus they have high values in the User Interface development features. However, it can be seen how they present important differences regarding their UI adaptation capabilities. With the more specific technologies, like MASP, Egoki or ICrafter including support to adapt the UI at run-time and automatically, and the more generic ones, like MARIA or UsiXML with manual support for adaptation.

Regarding Dandelion, thanks to its features to decouple developers from specific technologies and let them focus on user interface design, it scores high values on UI related features. Furthermore, as it has been designed with physical and tangible user interfaces in mind, it also has very nice support for different interaction modalities and heterogeneous interaction devices. Finally, Dandelion also provides good support for UI adaptation thanks to the GIP and the dynamic mapping between FIOs and the abstract UI, that allows an application to change from one UI setup to another at runtime.

Finally, the table in Figure 8 it is also a good indication of why we think that, apart from Dandelion, there is a lack of good enough solutions to build Physical User Interface under the Ubiquitous Computing constraints for distributed user interfaces with support to be used in very different interaction scenarios. While we have reviewed many solutions, only a few of them address more than one topic at the same time, with the majority of available solutions focused on supporting UI adaptation in the field of GUI and voice-based UIs. So, again looking at the table in Figure 8, those solutions that perform well on items in the left side of the table, perform poorly over those in the right side and vice versa. Only Dandelion, and to some extent iStuff, have some support for all the proposed aspects. Nevertheless, as shown in the table, iStuff is not so good at UI development because it is more focused on connecting devices than on supporting the development of distributed UIs. In particular, iStuff does not provide a unique standard interface between the UI and the devices, like the GIP in Dandelion, instead it requires developers to write some translation code to convert the particular remote interface of each iStuff device to the one required by each application.

### 4.2. Quantative Analysis

Dandelion represents a mixture of UI development and device control technologies clearly focused in fulfilling the requirements of building Distributed Physical User Interfaces for Ubiquitous Computing systems. As can be extracted from the state of the art review of Section 2 and the feature comparison from Section 4.1, there is very little literature focused on this particular niche, with the majority of the solutions available being technologies designed for other purposes that can be reused for building custom DPUIs.

Therefore, as we previously introduced in Section 3, nowadays, the usual way to build UIs for Ubiquitous Computing systems is to design and implement them ad-hoc, relying a combination of technologies coming from the HCI, IoT, home automation and distributed computing world. Thus, in order to provide a quantitative comparison between Dandelion and existing alternatives, we decided to perform a comparison using two objective development complexity measures: non commenting source code lines (NCSS) and number of public classes required to build two example systems using ad-hoc technologies and Dandelion. The number of public classes provides an indication of API and project complexity, and the NCSS provides an indication of code complexity and development effort. Regarding NCSS, we have ensured that all the code follows the same code style using the Checkstyle tool.

**Figure 9:**
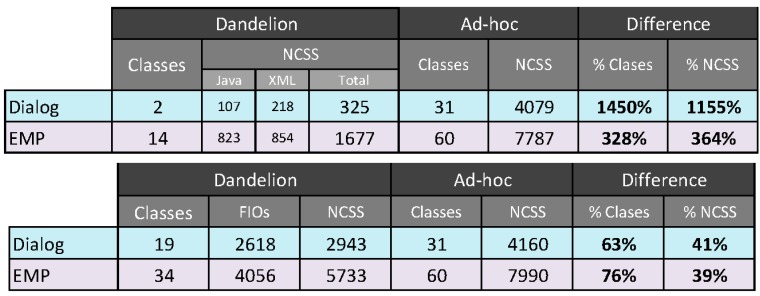
Comparison, in number of public classes and NCSS between developing two different UIs using Dandelion or using the different technologies directly. On the first table we are assuming that all the required FIOs are already available, on the second one we are also counting the effort required to implement all the FIOs required. The “Difference” column shows the % of increment in public classes and NCSS between using Dandelion or not.

Figure 9 shows the results of this comparison. We have used two examples of ubiquitous computing applications. The first one, Dialog, is one of the smallest possible example of a ubiquitous user interface. It can be seen as a standard dialog window in a GUI system that shows a message to user, and allows choosing between two different actions, either accept the message or reject it. The second one, EMP, is the Environmental Music Player used for the previous examples in this paper.

Regarding the Dialog example, we have designed and implemented the system for three different usage scenarios:

- First, a blind user, for whom we are using two different interaction modalities. Voice synthesis for outputting the dialog message, and hand and body gestures (using Microsoft Kinect) for the input of the user response.
- Second, a deaf user, for whom we are using three different interaction modalities. A graphical UI in a TV display for showing the message, a physical remote controller for the input of the answer, and the vibration of a smartphone, activated by the output of the message, to signal that there is a message to review.
- Third, we are reusing the TV display and the gesture modalities for a user without any disability.

In this case, without the abstraction capabilities of Dandelion, we have to rely in at least five different technologies, and we have to implement ourselves the software required to connect all of them in a distributed way. This is why, looking at the first table in Figure 9, there is a huge difference in effort between using Dandelion or not for the Dialog example. With Dandelion only 2 classes are required, one for loading the abstract UI and one for the UI behavior logic, and this is just 325 lines of code counting the XML code for the abstract UI definition and FIO mappings for the three examples. While in the case of building the UI in an ad-hoc way, we have to build three different UIs with five different technologies and connect them remotely (we have used a STOMP-based messaging system) which leaves us with 31 classes and 4079 lines of code, an increment of 1450% and 1155% respectively.

As can be seen, even a very simple example like a dialog window can get quite complex when we have to build it as a distributed UI adapted to the characteristics of different users and scenarios.

Nevertheless, it is important to note that in the results show in the first table of Figure 9, we are assuming that the developers already have all the FIOs required for the five different interaction devices. Dandelion has been designed to leverage the existence of FIOs for different technologies, while this is not always the case, especially the first time that a team uses Dandelion, the fact is that FIOs can be very reusable between different projects, and are easy to implement, as shown by the results of the second table in Figure 9, where we have added the number of public classes and NCSS of the different FIOs to Dandelion columns, and even with that, using the interaction technologies directly requires about a 41% more development effort. Similar results can be seen for the EMP case, where the benefits of using Dandelion are not so dramatic but still huge, the ad-hoc system implies an increment of 328% in the number of classes and 364% in NCSS. The difference with the Dialog example arises because we are not only counting UI code, but also system logic code, which in this case is much bigger than in the Dialog example.

## 5. Conclusions

In this paper we have provided an in-depth description of Dandelion, a software framework to facilitate the development of Ubiquitous Computing User Interfaces adaptable to different usage scenarios. This framework allows the implementation of user interfaces by declaratively describing its interaction requirements using a high-level abstraction model and implementing the UI logic on top of those abstract interaction components. This way, it makes the UI design and behavior completely independent of the final implementation. Furthermore, this complete isolation from the developers, their code, and the particular implementation of the UI, enables the deployment of very different realizations of the same UC user interface. Using Dandelion, the final UI implementation is not decided until deployment, thus it is possible to deploy different UIs in different scenarios.

Dandelion, as can be seen in the state of the art review of Section 2 and the comparison presented in Section 4, successfully improves the state of the art regarding important requirements of UC user interfaces, like UI distribution, UI abstraction and support Physical User Interfaces.

Finally, the comparison of Section 4.2 clearly illustrates that using Dandelion instead of relying in an ad-hoc mixture of technologies, can lead the huge reductions in the development efforts required to build UC user interfaces.

## Abbreviations

The following abbreviations are used in this manuscript:

DPUI	Distributed Physical User Interface
NUI	Natural User Interface
IMA	Interaction Modality Abstraction
ITA	Interaction Technology Abstraction
GIP	Generic Interaction Protocol
IR	Interaction Resource
UC	Ubiquitous Computing
AmI	Ambient Intelligence
